# Modelling the variation of land surface temperature as determinant of risk of heat-related health events

**DOI:** 10.1186/1476-072X-10-7

**Published:** 2011-01-21

**Authors:** Yan Kestens, Allan Brand, Michel Fournier, Sophie Goudreau, Tom Kosatsky, Matthew Maloley, Audrey Smargiassi

**Affiliations:** 1 Département de médecine sociale et préventive, Université de Montréal, Montreal, Canada; 2 Centre de recherche du Centre Hospitalier de l'Université de Montréal (CRCHUM), Montreal, Canada; 3 Direction de santé publique de Montréal, Montreal, Canada; 4 Institut National de Santé Publique du Québec, Montreal, Canada; 5 BC Center for Disease Control, Vancouver, Canada; 6 Natural Resources Canada, Ottawa, Canada; 7 Département de santé environnementale et de santé au travail, Université de Montréal, Montreal, Canada; 8 Chaire sur la pollution de l'air, les changements climatiques et la santé, Université de Montréal, Montreal, Canada

## Abstract

**Background:**

The evaluation of exposure to ambient temperatures in epidemiological studies has generally been based on records from meteorological stations which may not adequately represent local temperature variability. Here we propose a spatially explicit model to estimate local exposure to temperatures of large populations under various meteorological conditions based on satellite and meteorological data.

**Methods:**

A general linear model was used to estimate surface temperatures using 15 LANDSAT 5 and LANDSAT 7 images for Quebec Province, Canada between 1987 and 2002 and spanning the months of June to August. The images encompassed both rural and urban landscapes and predictors included: meteorological records of temperature and wind speed, distance to major water bodies, Normalized Differential Vegetation Index (NDVI), land cover (built and bare land, water, or vegetation), latitude, longitude, and week of the year.

**Results:**

The model explained 77% of the variance in surface temperature, accounting for both temporal and spatial variations. The standard error of estimates was 1.42°C. Land cover and NDVI were strong predictors of surface temperature.

**Conclusions:**

This study suggests that a statistical approach to estimating surface temperature incorporating both spatially explicit satellite data and time-varying meteorological data may be relevant to assessing exposure to heat during the warm season in the Quebec. By allowing the estimation of space- and time-specific surface temperatures, this model may also be used to assess the possible impacts of land use changes under various meteorological conditions. It can be applied to assess heat exposure within a large population and at relatively fine-grained scale. It may be used to evaluate the acute health effect of heat exposure over long time frames. The method proposed here could be replicated in other areas around the globe for which satellite data and meteorological data is available.

## Background

Heat waves and urban heat islands have been associated with increased mortality, particularly among persons with social or physical vulnerability [1-3]. With an increasing proportion of the world population living in urbanized regions, aging vulnerable populations, and climate change signalling increased frequencies of extreme climatic events and heat waves [4,5], the need to better understand ambient temperature determinants and associated health risks is important. Whereas the effect of elevated temperatures on human health has been widely studied over recent decades [6], epidemiological studies analysing acute health risks associated with exposure to elevated ambient temperature have mostly relied on meteorological stations to estimate temperature exposure. Yet, because meteorological stations are often located in sparsely inhabited areas - such as airports and parks - they provide only a partial representation of ambient temperatures in heterogeneous urban, suburban and rural landscapes where populations live [7]. Yet, variations in vegetation densities, open spaces, concrete surfaces, building disposition and height condition lead to micro-climatic variations in diurnal and nocturnal temperatures [7-9] within a same city [10,11]. As such, ambient air temperature measured at meteorological stations or simple interpolations between temperatures measured at stations may offer misleading measures of true exposure to local heat experienced in residential settings and so may bias the estimation of associated health risks. In support of this, we have shown that the risk of death associated with high daily temperatures at meteorological stations, was greater in areas with higher surface temperatures than in areas with lower surface temperatures [3].

Because the existing network of ambient temperature sensors only provides information at a few selected locations, remotely sensed thermal infrared (TIR) data has been used to estimate intra-urban variations in ambient temperatures, and to identify "hot spots" referred to here as "micro-urban heat islands". Various satellite or air-borne sensors including NOAA-AVHRR [12], MODIS [13] and LANDSAT [14], have been used to that purpose. Among these, the 60 m spatial resolution of LANDSAT 7ETM+ in the thermal IR band provides a useful resolution for analysing spatial variations in surface temperatures within cities, as well as along the urban-rural continuum.

Concordance between satellite-derived surface temperature estimates and ambient temperatures measured at meteorological stations can be strong in certain atmospheric conditions[15,16] (e.g. clear skies and mixing of any surface-based inversion layer[17]). This holds for various land cover configurations (accuracy to within ± 3-5% on average)[14,18,19]. Hence, surface temperatures derived from satellite images offer an interesting basis for establishing local measures of exposure to heat, and may be used in epidemiological studies of the association between exposure to temperatures and acute mortality or morbidity. However, satellite images are usually available for a limited set of dates, which prevent direct connection between daily vital statistics and satellite-derived land surface temperatures (LSTs). Furthermore, relations between meteorological measures and satellite-derived LSTs may differ depending on areas. Land-use regression techniques allow to model values of a spatially continuous phenomenon like air quality or surface temperature using local characteristics of the built environment. Early applications have modeled road salt contamination[20] or soil depths[21], but most applications concern the modelling of intra-urban variations in air pollution as a mean to improve precision in exposure for epidemiological models [22-24]. The aim of this paper is to develop a land-use regression of local surface temperatures using land cover, meteorological, and locational and temporal predictors, which can be used for both surveillance and for epidemiological analyses of acute heat-related population health outcomes over large territories.

## Methods

### Satellite Images

**Figure 1:**
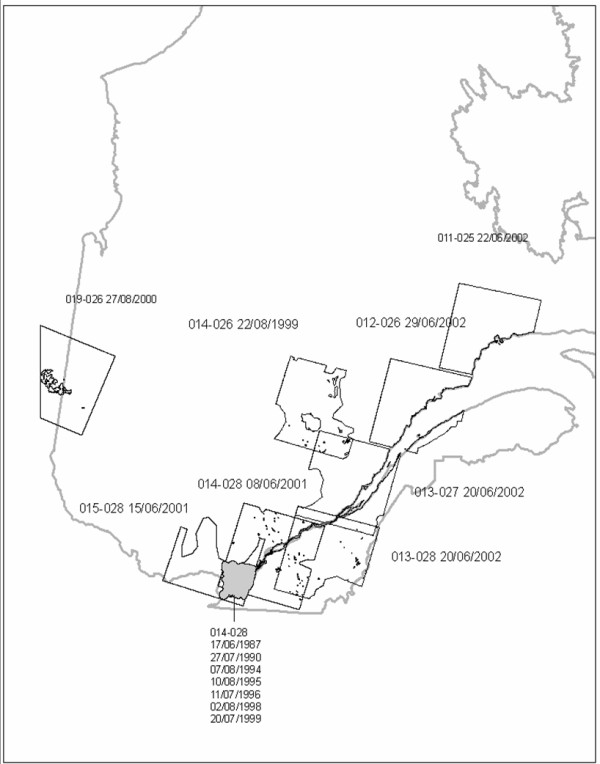
**Map indicating location of extents used in Quebec Province with dates of images used**.

We used a total of 15 LANDSAT multispectral images. Eight LANDSAT 7ETM+ images, taken between 1999 and 2002, covered different but partially overlapping extents in the southern portion of Quebec Province, Canada (See Figure 1: extents correspond to areas of roughly 200 km × 200 km, for which images provide 30 m cell-sizes for non-IR channels, and 60 m for IR channels). The regions covered, as listed below, included the main inhabited and urban areas of the province of Quebec: Montreal, Quebec City, Rimouski, Sept-Îles, Sherbrooke, Saguenay, Gatineau, and Rouyn-Noranda, representing some 90.3% of the total province population according to the 2006 Census (See Table 1 and Figure 1). In addition to these images, seven LANDSAT 5TM images, taken between 1987 and 1999, and covering part of the extent 014-028 over the greater Montreal region, were also used (cell size of 60 m for non-IR channels, and 120 m for IR channels); this extent selection covered urban, rural and suburban areas. Given the number of images used for part of this extent, our model best predicts surface temperatures for these regions, even if it extends to other inhabited regions of Quebec. All images where taken during June, July and August, under clear sky conditions, and contained less than 10% cloud coverage. Further hand removal of cloud pixels was done to retain only pixels representing surface temperature. The LANDSAT 7ETM+ image covering the Montreal region overlapped partly with the LANDSAT 5TM images of Montreal, but each image was treated separately. Image locations, dates, and times are given in Table 1. Given the varying spatial resolution of the different maps, pixels for all channels were re-sampled to 30 m, so as to keep the highest resolution when available.

**Table 1:** Satellite image data summary^1^

	Area	Extent	Date	Hour	Average surface temperature	Maximum surface temperature	Number of meteorological stations used
Landsat 5TM	Montreal	014-028	1987/06/17	10:00 am	19.33	55.26	2
	Montreal	014-028	1990/07/27	10:00 am	22.90	35.78	2
	Montreal	014-028	1994/08/07	10:00 am	20.24	56.29	4
	Montreal	014-028	1995/08/10	10:00 am	23.26	37.73	5
	Montreal	014-028	1996/07/11	10:00 am	18.58	31.39	5
	Montreal	014-028	1998/08/02	10:00 am	21.83	37.35	4
	Montreal	014-028	1999/07/20	10:00 am	22.03	41.94	5
Landsat 7ETM+	Montreal	014-028	2001/06/08	10:00 am	16.40	32.52	13
	Gatineau	015-028	2001/06/15	11:00 am	19.48	30.94	10
	Quebec	013-027	2002/06/20	10:00 am	16.57	31.20	10
	Rimouski	012-026	2002/06/29	10:00 am	16.61	27.71	9
	Rouyn-Noranda	019-026	2000/08/27	11:00 am	13.77	28.53	3
	Saguenay	014-026	1999/08/22	10:00 am	15.64	25.78	9
	Sherbrooke	013-028	2002/06/20	10:00 am	17.40	31.47	2
	Sept-Iles	011-025	2002/06/22	10:00 am	14.32	27.17	2

^1 ^Times are given using Eastern Standard Time

### Surface temperature calculation

LANDSAT images were processed using the automated cloud cover assessment (ACCA) algorithm [25]. We retained images with less than 10% cloud coverage. Remaining cloud-contaminated pixels were further removed by a research assistant using a GIS before the analysis.

Spectral radiance (R) was calculated from the LANDSAT thermal channel digital numbers (DN), using gain and bias values included in the LANDSAT image product according to:

R=G(DN)+B

where G (gain) and B (bias, or offset) are calibration values included in the LANDSAT image product. Radiance values of all pixels were then converted to surface temperature (T) values according to:

$$LST=\frac{K_{2}}{\ln(\frac{K_{1}}{R}+1)}$$

where K_1 _is 607.76 for TM and 666.09 for ETM+ and K_2 _is 1260.56 for TM and 1282.71 for ETM+ [14].

### Predictors of calculated surface temperatures

#### Land cover

Land cover types were determined using the maximum likelihood algorithm provided by PCI Geomatics, with a supervised classification scheme using all channels except the thermal IR band (band 6). Three land cover categories were defined: built and bare land, vegetation, and water. Stable sites, such as the Olympic stadium and Botanical Gardens in Montreal, were used as reference points for land cover specification while controlling for inter-annual variability in images.

#### NDVI

The Normalized Differential Vegetation Index was calculated based on LANDSAT bands 3 (Red) and 4 (Near-Infrared) according to standard practice[26]. Using images taken between the first week of June and the last week of August allowed us to limit inter-seasonal variation in vegetation density and chlorophyll activity throughout the summer season.

#### Meteorological data

Meteorological data from all available Environment Canada stations [27] providing hourly data within each extent was simply averaged, to obtain image-specific measures of ambient temperature and wind speed at the time the images were taken as well as for the three-day average, that is, for the day the image was taken and the two previous days. This simple approach was used with the view of easily reproducing the method in future model use. Between two and fifteen meteorological stations with complete hourly data were available per image (see Table 1).

#### Complementary spatial and temporal measures

Because proximity to large water bodies is known to influence LST [28], the Euclidian distance to the nearest of three main water bodies in Quebec (St. Lawrence River, Saint-Jean Lake, and Abitibi Lake) was computed for each pixel. Latitude and longitude was derived within a projected Universal Transverse Mercator coordinate system (UTM, NAD83 zone 19N). A dummy variable relating to each image was further used to capture any possible inter-image variations not accounted for by the other predictors. Week number was used to account for temporal variation.

### Statistical analyses and validation

We used a random subsample of 2% of the total number of pixels (*n *= 480,000 points) done to reduce the large number of total pixels of the 15 images in our analysis (n = 246,590,536). Within each map, the number of points sampled was proportional to the area. Using GIS, we computed LST and predictor data for each of the 480,000 sampled 30 m resolution pixels.

A general linear model (GLM) was developed to estimate land surface temperatures of the sampled pixels using land cover, NDVI, meteorological data and complementary spatial and temporal information described above (see Formula 1).

(1) $$\begin{array}{l} LST=M\beta_{m}+LC\beta_{lc}+XY\beta_{xy}\\ +T\beta_{t}+E\beta_{e}+\varepsilon \end{array}$$

With M being a matrix of meteorological indicators (air temperature, wind speed), LC a matrix of land cover indicators (land cover categories and NDVI), XY a matrix of latitude longitude indicators, and T a matrix of temporal indicators (Week number in our case), E a matrix of extent indicators, all Betas being vectors of corresponding regression coefficients, and epsilon the error term.

Inter-variable correlation was tested using Pearson's correlation coefficient, whereas spatial autocorrelation (SA) of residuals was assessed using Moran's I to evaluate possible unexplained spatial variation in surface temperatures. The minimum distance considered for SA was 120 m, the size of the IR channel cell in LANDSAT 5. We calculated SA for each map separately and present the average for all maps. The predictive ability of the model was evaluated by applying the model to the whole dataset (n = 246,590,536) and computing the standard error of the estimate.

## Results

From the 480,000 sampled points, 8 points with negative surface temperature value were removed and an additional 298 points were excluded due to edge effects on the maps or missing data in one or more other layers. The remaining 479,694 points were used to build the model. The surface temperature of the sampled points had a similar distribution to those of the complete dataset, with a slightly higher mean value, that is, 16.9°C (SD = 2.97, Min = 6.2°C, Max = 37.3°C) compared to 16.6°C (SD = 3.02, Min = 6.2°C, Max = 37.4°C) for the whole sample. Table 2 presents descriptive statistics of the explained and predictive variables for the subsample of points used to build the model with the 15 images. Land cover classification resulted in characterising 73.51% of pixels as vegetation, 22.87% as built or bare land, and 3.60% as water surface.

**Table 2:** Descriptive summary of surface temperatures, meteorological data, and time and location information for the 15 images covering parts of Quebec Province

	Min	Q1	Median	Q3	Max	Pixel level (P, n = 479694), or image level (I, n = 15)
Surface T°	6.2	15.08	16.29	18.37	37.35	P
Ambient T°	10.85	17.27	19	20.2	31	I
3-day mean T°	14.71	15	16.86	17.92	23	I
Wind (km/h)	0.8	6.2	7.33	10.88	15	I
Normalised Differential Vegetation Index	-0.83	0.21	0.38	0.5	0.82	P
Week No.	22	24	24	31	35	I
Distance to Water (m)	0	10642	36192	66104	148202	P
X (km)	-852.28	-392.48	-257.66	-140.72	241.12	P
Y (km)	0.00	233.74	340.15	576.88	804.90	P

	Water	Built/Bare	Vegetation			

Land Cover (proportion of land use per category)	3.60%	22.87%	73.51%			P

Inter-variable correlation tended to be relatively weak (*r *= 0.27, SD = 0.07). The highest correlation was found between week number and wind speed (*r *= 0.54). LST correlations between overlapping images ranged from 0.212 to 0.685.

The GLM explained a relative high proportion of the variation in surface temperature (*R² *= 0.77), and resulted in a relatively low residual standard error (1.42°C; Table 3). Predicted temperatures ranged between 7.9 and 29.0°C.

**Table 3:** Parameter coefficients of multiple regression model predicting Land Surface Temperature within 15 Landat Images in populated areas of Quebec Province between June and August, 1987-2002

		Coefficient	St. Error	*t*
Parameters	Intercept	0,53	0.81	6.59
	Ambient T°	-0.061	0.04	-17.19
	3-day mean T°	0.557	0.04	148.14
	Wind (km/h)	-0.075	0.01	-52.10
	Built and Bare Land	5.884	0.13	45.58
	Vegetation	5.542	0.14	40.44
	NDVI	-5.541	0.13	-42.62
	Week No.	0.324	0.02	145.29
	Distance to Water (1000 km)	-0.011	0.001	-148.45
	X (1000 km)	-0.08	0.004	-187.14
	Y (1000 km)	-0.27	0.044	-62.27
				
Extents	Outer Montreal	-1.05	0.02	-66.13
	Gatineau	-2.09	0.03	-67.44
	Quebec	-0.29	0.02	-12.65
	Rimouski	-0.24	0.02	-9.69
	Rouyn-Noranda	-7.58	0.04	-188.83
	Saguenay	-5.32	0.02	-250.24
	Sherbrooke	-0.43	0.02	-21.44
	Sept-Iles	-3.40	0.03	-102.92
Number of observations: 479694	Adjusted R-Square: .77	SE: 1.42°		

NB: All variables significant at p < 0.0001

Coefficients for model parameters are given in Table 3. All predictors were significant at the 0.0001 level. Overall, when comparing the entire data ranges of the predictors, it was found that NDVI had the strongest effects, with a 95% effect range of 9°C - or, in other words, 95% of the variation in NDVI explained up to 9°C variation in surface temperature. Land cover, week number, three-day average temperature, and our dummy map predictor also showed large effect ranges, with 95% effect ranges of 5°C. Other parameters tended to have smaller effects.

### Model Analysis and Validation

Spatial autocorrelation among surface temperatures was high, and significant up to a 2 km range, beyond which SA decreased significantly. However, the Moran's I values for the model's residuals were significantly reduced, but remained significant in the 0-2 km range, suggesting additional environmental factors would need to be accounted for to fully explain variations in LST. As mentioned earlier, spatial autocorrelation does not influence our prediction of surface temperatures. A closer look at local spatial autocorrelation may be useful to raise new hypotheses identifying additional relevant environmental determinants.

**Figure 2:**
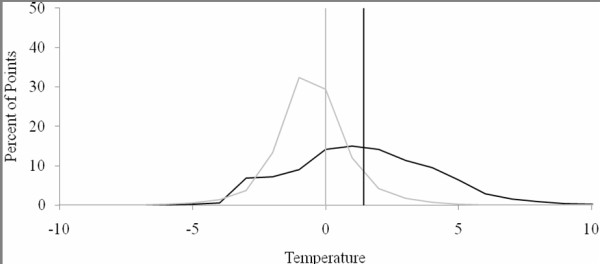
**Residual distributions of 2% modelling sample (grey) and 98% validation dataset (black)**. Mean values shown by vertical lines.

Estimates of surface temperatures were comparable to observed LANDSAT-derived point data. Figure 2 shows the distribution of residuals of the point data. The predictive capabilities of the model was evaluated by applying the model to the whole dataset (n = 246,590,536) and comparing predicted to observed values. The mean difference between observed and predicted temperatures was 1.42°C (Standard deviation = 2.67).

## Discussion

This paper presents a statistical model of surface temperatures computed with some 15 satellite images taken during the 1987-2002 period covering the main inhabited areas of Quebec Province. Using basic meteorological, land cover, and time predictors, this model allows local estimation of surface temperatures in the most populated areas of Quebec for the three warmest months of the year. Such a model is particularly useful because existing observed ground temperatures are only available for specific locations - that is, where meteorological stations are installed - and satellite derived temperature readings are only available for specific dates. In the context of land use and climate change, such a model provides local estimates of surface temperature that can be used in epidemiological studies on acute heat-related health outcomes, and as a planning and prevention tool.

As expected, meteorological indicators of air temperature, especially the three day average, were strong predictors of LST. Also consistent with the literature, land cover predictors (i.e. NDVI and land cover categories) were significantly and strongly associated with surface temperatures [29-31]. The cooling impact of NDVI has been well documented [29,30], and variations in LSTs within urban areas have been linked to the spatial configuration of land use and land cover [30,31].

Application of surface temperature estimates to epidemiological models of heat-related health outcomes has often been limited by the availability and the relatively low resolution of surface temperature maps that cover large areas. The use of a spatio-temporal model is therefore advantageous as it allows matching between high spatial and temporal resolutions of temperature estimates and at-risk populations or health events. By providing surface temperature estimates for any location and time, it overcomes the potential temporal and spatial discrepancy between either available images or available meteorological data and health outcome events. Meteorological data has usually been used as a proxy of heat conditions across a large region, with temperature from such stations either considered uniform or simply interpolated across an extent [7]. Because our model explicitly integrates spatial terms, like land-cover and greenness, micro-variations in temperatures - that is, differences at the urban lot size scale - can be explored. The use of a relatively large set of images - covering most of the inhabited areas of the Quebec province, including summer's intra-seasonal variations - allows for the creation of calibrated models across the province. These models are representative of June to August summer days, but were not specifically calibrated for extreme heat event conditions. Further work is needed to assess the robustness of this model for prediction of surface temperatures under extreme heat events.

There are other limitations inherent to both the model and to the use of satellite imagery for our capacity to predict LSTs.First, our outcome measure, LST, can be calculated in different ways, and may for example account for emissivity or other parameters. Here we used the simplest but classic and validated formulae for LST determination [14]. The effect of downscaling to a resolution of 30 m on the association between LST estimates and health outcomes deserves further attention. The model itself can be improved by refining or better describing physiographic features of the landscape. For instance, elevation or orientation was not considered in our model, although the use of such measures for surface temperature modelling is relatively common [32]. However, studies using satellite imagery to examine surface temperatures have not explicitly looked at the relation with elevation or orientation [29,33]. One of the limitations to such data integration relates to the resolution of available digital elevation models (DEM), especially when covering such large areas. We only had access to a coarse Canadian-wide DEM providing a 100-point per latitude/longitude elevation matrix (10 points of latitude * 10 points in longitude). Use of more fine-grained DEM may improve the performance of our model.

The land cover classes used were also relatively general. Although coarse classification does limit our capacity to assess micro-level variations in LST, because local structural differences within land cover classes are not accounted for, the use of broad categories does reduce the risk of misclassification. It has been documented that areas of urban-rural transition are especially prone to such classification errors [34], and the potential benefit of increasing classification precision, with an associated risk of misclassification, is still unclear. Yet, interesting novel methods suggest possible increased precision in land cover classification based on TM images, with possibility to discriminate residential from commercial and industrial land, account for density, and specifically identify urban green space[35].

The fact that significant residual spatial autocorrelation remained in our models, suggests that further addition of spatial predictors would be required to improve the predictive capacity of the model and limit coefficient bias. As an example, integrating wind directions may be useful to account for the cooling effect of water bodies in downwind areas[28]. Alternatively, explicit spatial modelling strategies, like inclusion of autoregressive terms or use of Geographically Weighted Regression models for example, could be used. "When obtaining point predictions, it matters little if we model the spatial variation entirely through the covariates, entirely as small-scale variations characterized by the semivariogram or sigma, or through some combination of covariates and residual autocorrelation. Our choice of covariates mainly affects the interpretation of our model and the magnitude of the prediction standard errors" [36].

While our model does predict outdoor surface temperatures, heat exposure that may be relevant for epidemiological models would need to account for both outdoor and indoor temperatures. As such, remotely sensed surface temperatures provide only a fraction of true exposure, and complementary analysis using building characteristics may be used to further provide estimates of indoor exposure to heat. Previous work on the subject revealed a relatively linear relation between indoor and outdoor surface temperatures, with a positive influence of building height [37], for dwellings that were not equipped with air conditioning.

Limitations in the use of satellite imagery for establishing models of land surface temperatures are also inherent to the absence of precise surface geometry information. Surface geometry of buildings can induce strong micro-urban temperature patterns due to differential solar heating [38-41]. An associated directional bias is discussed by Nichol [39], where near-nadir view-angle of satellite sensors may yield temperatures that are warmer or cooler than off-nadir views, depending on the view direction relative to solar position, time of day, and surface characteristics of the environment such as building disposition and height. While the effect of surface geometry on temperature was not considered in our study, its effects can be pronounced and could be considered in future developments. Lagouarde et al. [40] have reported temperature differences upwards of 12°C between nadir and off-nadir temperatures in Marseille, France, while Voogt and Oke [41] found a maximum difference of 10°C in Vancouver, Canada. The mid-morning LANDSAT images used in our study may be prone to this bias as rooftops heat up much faster than shaded walls do, because the low angle of morning sunlight offers potential for sizeable variation between nadir and off-nadir temperatures [39].

Additionally, our model best predicts surface temperatures for the years for which the images used. As with other satellite based studies, the availability of adequate images constrains the utility of our model. Although sensors that produce NDVI and land cover (such as IKONOS, QuickBird, SPOT, or MODIS) are still widely available, there are currently few sensors with sufficient resolution Thermal IR capabilities for detailed micro-urban temperature mapping. Both LANDSAT 5 and LANDSAT 7 satellites are well past their mission's expectancy and the next satellite in the LANDSAT series, the LANDSAT Data Continuity Mission (LDCM), scheduled to launch in December 2012, is not yet expected to include a thermal IR imager [42].

Finally, precise air temperature estimates are only useful if corresponding health events can be mapped accordingly. Precise information on people's exposure locations are required to conduct such analyses. Information on peoples' main locations may further improve our capacity to unravel the effect of environmental dimensions on health[43].

## Conclusions

This type of land-use regression model optimises the spatial and temporal estimation of LSTs. Results from Smargiassi et al. [3] suggested that the risks of mortality associated to high temperatures is higher within micro-urban heat islands, over Montreal city. Another study held in Philadelphia looking at a heat event in July 1993 demonstrated the significance of remote-sensing land surface temperatures readings in predicting mortality, beyond socio-demographic information [44]. This model will provide estimates of exposure to heat islands within the inhabited regions of the province of Quebec, and improve our ability to explore environmental determinants of heat-related health events. Use of such models allows identifying features associated with heat that are amenable to change, like NDVI or land use. Using epidemiological models, cities may identify high-risk areas with low vegetation indexes, and implement policies to change the built environment. Furthermore, because it is possible to cover large and diversified territories, such an approach allows studying health risks in a variety of landscapes and urban configurations. With satellite, land use and meteorological data available in most populated settings, such models could be calibrated elsewhere to improve our understanding of the relation between heat events, land use, and acute heat-related health outcomes.

## Competing interests

The authors declare that they have no competing interests.

## Authors' contributions

YK finalised the manuscript and participated in the design of the study, the modelling, and interpretation of results. AB drafted the manuscript and performed certain statistical analyses. MF performed statistical analyses and helped interpret results. SG compiled geographic data and assisted in the modelling. TK and MM assisted in the design of the study and reviewed the manuscript. AS conceived the study, participated in its design and coordination. All authors have read and approved the final manuscript.
